# The influence of heatwaves on traffic safety in five cities across Québec with different thermal landscapes

**DOI:** 10.1186/s40621-025-00564-2

**Published:** 2025-02-28

**Authors:** José Ignacio Nazif-Munoz, Vahid Najafi Moghaddam Gilani, Juwel Rana, Ernani Choma, John D. Spengler, José Guillermo Cedeno-Laurent

**Affiliations:** 1 Faculté de Médecine et des Sciences de la Santé, Université de Sherbrooke, 150, Place Charles-Le Moyne, Longueuil, QC J4K 0A8 Canada; 2 Department of Epidemiology, And Occupational Health, Mcgill University, Mcgill College, Suite 1200, BiostatisticsMontreal, QC H3A 1G1C Canada; 3 Research and Innovation Division, South Asian Institute for Social Transformation (SAIST), Panthapath, Dhaka-1205 Bangladesh; 4 Department of Environmental Health, Harvard T.H. Chan School of Public Health, 401 Park Drive, 4 Floor West, Boston, MA 404N02215 USA; 5 Rutgers School of Public Health, Rutgers University, 170 Frelinghuysen Rd 23, Piscataway, 08854 USA

## Abstract

**Background:**

This study assesses the impact of heatwaves on road safety in five Québec cities (Montréal, Québec City, Laval, Longueuil, and Sherbrooke) from June to September 2015–2022. Using Urban Heat Island (UHI), 90th and 95th percentile thresholds for heatwaves, we analyze their effects on collisions, traffic injuries, and killed and seriously injured (KSI) cases.

**Methods:**

Traffic data were analyzed across two heatwave definitions, utilizing a time-stratified case-crossover design. UHI was approximated using the annual maximum of mean warm-season land surface temperatures (LST) derived from Landsat 8 (30 m resolution) over three consecutive years, identifying areas that stay hotter during the day and radiate excess heat at night. Heatwaves were defined as periods of at least two consecutive days with mean temperatures exceeding the historical 90th or 95th percentile of mean temperatures. Negative Binomial regression models were used to examine associations between UHI, heatwave events and traffic incidents. Models controlled for time varying variables such as rainfall, seasonality, and COVID-19 impacts.

**Results:**

Heatwaves, particularly at the 95th percentile threshold, significantly increased traffic incidents in Montréal and Longueuil. In Sherbrooke, the 90th percentile threshold showed significant effects on collisions and injuries, while Québec City and Laval exhibit no significant associations. UHIs show a modest overall increase in collisions (IRR: 1.07) but limited effects on traffic injuries and KSI. Differences across cities highlight Montréal's higher IRR for collisions under heatwaves and lower IRR for KSI compared to Québec and Longueuil respectively.

**Discussion and conclusion:**

The results indicate that cities like Montréal and Longueuil, with slightly stronger UHI and higher susceptibility to heatwaves, face increased road safety risks. However, UHI levels in Montréal were not significantly different from those in other cities, and heatwaves at the 95th percentile showed variability across regions. These findings highlight the need for targeted climate-adaptive strategies, such as green spaces and heat-reflective materials, to mitigate risks. Integrating climate resilience into urban planning remains critical as extreme weather events grow more frequent.

**Supplementary Information:**

The online version contains supplementary material available at 10.1186/s40621-025-00564-2.

## Introduction

Sudden changes in meteorological conditions can positively or negatively influence road safety outcomes [34]. While weather conditions can directly affect the occurrence of traffic crashes and injuries (TCI), they can also indirectly influence these outcomes by modifying mobility patterns [1, 12]. Previous research has explored climatological elements like ambient temperature, rainfall, and snowfall, among others, as explanatory factors in predictive models assessing risk factors for road traffic crashes [27]. More specifically, evidence indicates that fluctuations in temperature, rain, and snow increase the risk of TCI [37, 47]. However, although exposure to environmental conditions significantly impacts the incidence of TCI, their effects may vary based on other factors such as population density, road type, and vehicle speed [2].

Within this body of observational studies, the relationship between temperature and road crashes has consistently shown a positive correlation. One meta-analysis focusing on this relationship found that daily mean temperatures are associated with a 2% increase in road traffic crashes and a 5% increase in road traffic injuries [27]. Regarding high temperatures, findings are more mixed. One study found that high nighttime temperatures could be associated with a 30% increase in traffic fatalities in Santo Domingo, Dominican Republic. In contrast, no effects on injuries or fatalities were observed in Boston, United States [31]. Similarly, in Los Angeles, high temperatures were linked to increased road crashes, whereas in Chicago, no relationship between these two variables was observed [17]. Across the continental U.S., another study showed a significant positive association between fatal traffic crashes and heat waves, reporting a 3.4% increase in fatal crashes on heat wave days compared to non-heat wave days [44]. Similarly, in Spain's Catalonia region, the estimated crash risk increased by 2.9% during heat waves [3]. In Italy, for the years 2013–2015, traffic crashes rose by 12% on hot days [3].

Some studies have explored pathways that could explain the potential link between high temperatures and road crashes. These pathways suggest that elevated temperatures may cause road users to experience greater fatigue, reduced reaction times, adverse health effects, and/or more aggressive driving behavior [25]. Specifically, research on the impact of ambient temperatures has shown that high temperatures can impair driving performance by increasing drowsiness [45], irritability, and aggression [41], and/or slowing reaction times [11]. In non-driving conditions, the association between high temperature and slower reaction times has been documented in numerous studies [14, 24, 42].

A significant portion of studies examining the relationship between temperature and traffic outcomes has focused on the direct association between these two variables [4, 27, 28], rather than considering acclimation as an important but often unobserved factor linked to temperature changes and, consequently, traffic outcomes. Following Wu et al’s [44], we use heatwave definitions based on the 90th and 95th percentiles of historic temperature data to indirectly capture local acclimatization to heat. These thresholds help account for varying levels of heat intensity and frequency that populations are likely exposed to, reflecting how people might adapt to heat over time. Our definition of heatwaves differs from the official one used by Quebec authorities, which defines a heatwave based on regional Tmax and Tmin thresholds for at least three consecutive days [23]. Using these heat exposure metrics, we examine their associations with traffic outcomes: crashes, injuries, and fatalities.

Our study focuses on five cities within Québec—Montréal, Québec City, Laval, Longueuil, and Sherbrooke—selected for their robust road safety systems and variations in urban heat island (UHI) effects. UHI, characterized by elevated urban temperatures due to heat absorption by buildings and pavements, varies in intensity across these cities. Montréal and Longueuil exhibit slightly stronger UHI effects than Québec City, Laval, and Sherbrooke [8, 35, 46]. These variations in UHI may contribute to differences in traffic outcomes, as regions with more severe UHI conditions may experience prolonged or more intense exposure to extreme heat, potentially exacerbating the physiological and behavioral impacts of heat on road users. Previous studies on heatwave-related mortality and morbidity in Québec [7, 9, 26] highlight the elevated risks posed by extreme temperatures, which are often amplified in urban environments with stronger UHI effects. By integrating these findings with our analysis of traffic outcomes, we aim to provide a more comprehensive understanding of how heatwaves specifically affect road safety in cities with varying thermal conditions.

Our research goes further by examining how UHI and heatwaves interact with road safety in cities that share similar infrastructure and road safety programs. This nuanced approach allows us to explore the impact of extreme heat not only on general health, as documented in studies by Boudreault et al. [5] and Bustinza et al. [6], but also its specific influence on traffic-related injuries and fatalities in Québec. By accounting for both acclimation and UHI intensity, we aim to offer valuable insights into how heatwaves amplify the risks faced by road users. Ultimately, our study seeks to raise awareness of the heightened vulnerability of road users during extreme heat events, encouraging a more integrated approach that bridges environmental health with road safety strategies—an area that has seen limited data on traffic-related causes in Québec.

## Methods

### Study design

This study evaluated the impact of heatwaves on traffic crash outcomes across five highly populated cities of the province of Québec, Canada: Montréal (1,780,000), Québec City (542,298), Laval (437,413), Longueuil (246,855), and Sherbrooke (167,162) (maps pf each city can be found in the supplementary material). The analytic sample included all traffic crashes, injuries, and fatalities reported between June 1st and September 30th of each year from 2015 to 2022.

### Data

Daily data on traffic crashes, injuries, and fatalities were obtained from the *Société de l'assurance automobile du Québec* (SAAQ) [36]. This information is collected by police forces at the time a motor vehicle crash is reported and was processed and assigned causes of traffic crashes by the SAAQ based on the police reports. Population information was sourced from the *Institut de la statistique du Québec* [20]. Meteorological data from multiple meteorological stations operated by Environment Canada [15] (the list of stations per city can be found in the supplementary material). Additionally, information to control for non-pharmaceutical COVID-19 interventions implemented during the 2020–2022 period was considered from the QC-nP-COVID-19 index [29]. The data used in this study were publicly available, with the exception of SAAQ data, which requires specific authorization following Law 25. Therefore, ethical approval from the Université de Sherbrooke ethics committee was not required.

### Outcome variables

Three outcome variables were analyzed: (i) the number of car crashes, irrespective of injury type; (ii) the total number of light traffic injuries; and (iii) the total number of killed and severely injured (KSI) individuals. We also used the administrative region identification code of the crash location to create a binary indicator that flagged all crashes occurring within the confines of the designated urban conglomerates. Rates per 100,000 population were calculated for all three outcomes: all crash injuries, encompassing pedestrians, passengers, drivers of motor vehicles, and bicyclists, and all crash injuries and fatalities.

### Urban Heat Island

As a proxy for UHI, we used the three-year average annual maximum of mean warm-season land surface temperatures (LST) between April 1st and September 30th derived from Landsat 8 (30 m resolution). It identifies areas that stay hotter during the day and radiate excess heat at night, which contributes to the formation of heat islands in urban areas [7, 39]. CANUE developed this measure at the postal code level for Canada using a publicly available algorithm applied through Google Earth Engine [13]. We extracted the UHI associated with each city, representing area-weighted average values across those areas over time [8].

### Heatwaves

This study employed two definitions of heatwaves: (i) a daily mean temperature above the 90th percentile threshold for two or more consecutive days and (ii) a 95th percentile threshold for the same duration, based on Cheng et al. [21]. The definitions were applied using data in each selected city and matched to the crash date. For each year, the period of analysis to obtain these values was from June 1st to September 30th.

### Control variables

To properly assess the impact of heatwaves, we considered the Québec Non-Pharmaceutical Interventions Index (QCnPI-Index), rainfall and time. The QCnPI-Index, derived from various sources, encompassed 58 interventions categorized into three key areas—Travel, Physical distancing, and Closure/Opening—across the five studied cities. A higher score on the QCnPI-Index reflected a greater implementation of interventions, indicating a more comprehensive approach to limiting COVID-19 spread, while lower scores suggested a less comprehensive approach. We included this index to control for potential known confounding effects, as the literature has consistently shown significant reductions in crashes, traffic injuries, and fatalities following the introduction of multiple non-pharmaceutical interventions during the COVID-19 pandemic (Nazif-Munoz et al. 2024). Additionally, rainfall was considered as a time-varying factor influencing traffic outcomes during heat events. This variable is reported as millimeters of precipitation per day.

### Statistical analysis

In this study, a time-stratified case-crossover design [40] was employed to investigate the association between UHI, heatwaves and traffic crashes, injuries, and fatalities per population during June–September of 2015–2022. This design is widely utilized in environmental health studies for analyzing short-term exposure effects on acute health outcomes (Nhung et al. 2017). The case-crossover design is a variant of the case–control study, where each case serves as its own control, effectively controlling for individual-level confounding factors that remain constant over time. This approach is particularly suitable for investigating transient exposures such as heatwaves and their immediate effects on traffic safety outcomes. Specifically, the time-stratified case-crossover design divides the study period into strata of fixed length, typically days or weeks. For each case day (i.e., days with traffic crashes), one or more control days are selected from the same stratum, typically matching on the day of the week, month, and year to account for potential confounding by seasonal and long-term trends [43]. Negative Binomial regression is then applied to estimate the incidence rate ratios (IRR) and their corresponding confidence intervals. It quantifies the association between heatwave exposure and the risk of traffic crashes, injuries, and fatalities. The Negative Binomial regression model can be represented by the following Eq. (1):

1 $$\begin{aligned}\text{log}\left(E[Y]\right)&={\beta }_{0}+{\beta }_{1}\times Urban Heat Island + {\beta }_{2}\times Heatwave \\ & \quad+{\beta }_{3}\times Covariates +\text{log}(Population) \end{aligned}$$

where:

Y = represents the counts of traffic crashes, light injuries, or KSI occurring on a given day,

${\beta }_{0}$ is the intercept term,

${\beta }_{1}$ represents the coefficient of interest for the association between UHI,

${\beta }_{2}$ represents the coefficient of interest for the association between heatwave exposure,

${\beta }_{3}$ represents coefficients for additional covariates (rainfall, time, and the QCnPI-Index) included in the model,

Log (Population) serves as the offset to account for population size.

For sensitivity analyses, we used the Poisson function. The results, robust to our preferred model specification, are presented in the supplementary material (Tables S1 and S2). We selected Negative Binomial models, as the Deviance and Pearson tests indicated a better fit for addressing overdispersion (Tables S3 and S4). We also considered the potential lag effect in our analysis by including lags of up to seven days for each variable of interest. However, including these lagged variables resulted in a higher Bayesian Information Criterion than the model without lags, indicating a poorer model fit. Results were also robust to this specification (results are available upon request).

We employed a two-stage design to examine heterogeneity in the variables of interest across the five cities. First, the results from each regression, as described in Eq. (1) for each city, were pooled for the overall effects of UHI and heatwaves using an extended random-effects meta-analysis, following the methodology proposed by Sera and Gasparrini [38]. Cochran’s Q and the I^2^ statistic were used to evaluate heterogeneity in the associations across cities: Q measures each study’s deviation from the pooled estimate by weighting its contribution through inverse variance, whereas I^2^ represents the proportion of total heterogeneity attributed to between-city differences (I^2^ < 25% ~ low heterogeneity) [22]. Additionally, we conducted a Wald test and calculated the corresponding Z-scores for comparison, as Hoffmann et al. [16] outlined, to investigate whether the incidence rate ratios (IRRs) differed significantly between cities.

## Results

### Urban Health Island and temperature

Table 1 summarizes the UHI results for each city in 2015 and 2022 and the number of heatwave days at the 90% and 95% temperature thresholds, along with the average, minimum, and maximum temperatures for the full analysis period (2015–2022). Montréal experienced 50 heatwave days at or above the 90% threshold and 11 heatwave days at the 95% threshold. UHI data revealed that Montréal’s average UHI was 26.68 °C in 2015, rising to 30.17 °C in 2022. Montréal, and possibly Longueuil, exhibited the highest UHI values across the cities studied, with Longueuil’s UHI rising from 26.67 °C in 2015 to 30.18 °C in 2022. In comparison, Québec’s UHI increased from 23.92 °C in 2015 to 29.65 °C in 2022. This city also recorded the most days at the 90% threshold (72) and had a relatively high count at the 95% threshold (18). Laval displayed a similar pattern to Longueuil, with 65 days at the 90% threshold and 28 at the 95%, and an increase in UHI from 26.30 °C in 2015 to 29.42 °C in 2022. Sherbrooke’s UHI showed the least change, rising from 24.04 °C in 2015 to 26.59 °C in 2022. Sherbrooke experienced the highest overall number of heatwave days, with 75 days at the 90% threshold and 42 at the 95% threshold. Regarding average temperatures, Montréal showed the highest mean temperature (20.1 °C), followed closely by Longueuil (19.5 °C), while Québec and Sherbrooke had the lowest averages (17.6 °C). The highest maximum temperatures were observed in Montréal and Longueuil, each exceeding 36 °C, whereas Québec and Sherbrooke had slightly lower peaks, reaching 33.6 °C. In terms of minimum temperatures, Sherbrooke recorded the lowest at −4.1 °C, followed by Québec (−2.7 °C) and Laval (−1.7 °C), while Montréal had the warmest minimum at 2.0 °C.

**Table 1:** Summary of weather variables and control days by city, including heatwave temperature thresholds, June through September 2015–2022

**City**	**Weather variables**	**Number of days**	**Number of control days**	**Mean (°C)**	**Min (°C)**	**Max (°C)**	**Heatwave 90% Max Temperature (°C)**	**Heatwave 95% Max Temperature (°C)**
**Montréal**								
	***Urban Heat Island 2015***			26.68	18.95	29.97		
	***Urban Heat Island 2022***			30.17	17.81	35.23		
	***Heatwave 90%***	50	926				36.1	
	***Heatwave 95%***	11	965					35.3
	***Temperature***			20.1	7.9	30.1		
	***Max temperature***			25.0	12.1	36.1		
	***Min temperature***			15.1	2.0	24		
**Québec**								
	***Urban Heat Island 2015***			23.92	16.29	27.49		
	***Urban Heat Island 2022***			29.65	21.08	34.74		
	***Heatwave 90%***	72	904				33.6	
	***Heatwave 95%***	18	958					33.6
	***Temperature***			17.6	4.4	27.4		
	***Max temperature***			23.2	10.9	33.6		
	***Min temperature***			11.8	−2.7	22.6		
**Laval**								
	***Urban Heat Island 2015***			26.30	19.52	29.79		
	***Urban Heat Island 2022***			29.42	19.98	34.02		
	***Heatwave 90%***	65	911				34.4	
	***Heatwave 95%***	28	948					
	***Temperature***			18.2	4.8	27.2		34.2
	***Max temperature***			23.3	11.1	34.4		
	***Min temperature***			12.9	−1.7	22.7		
**Longueuil**								
	***Urban Heat Island 2015***			26.67	18.84	29.99		
	***Urban Heat Island 2022***			30.18	19.45	34.92		
	***Heatwave 90%***	68	908					
	***Heatwave 95%***	34	942				36.2	
	***Temperature***			19.5	7.4	28.7		36.2
	***Max temperature***			25.0	12.1	36.2		
	***Min temperature***			13.9	−1.0	23.9		
**Sherbrooke**								
	***Urban Heat Island 2015***			24.04	17.50	29.15		
	***Urban Heat Island 2022***			26.59	20.84	30.28		
	***Heatwave 90%***	75	901				33.6	
	***Heatwave 95%***	42	934					33.6
	***Temperature***			17.6	3.7	30.5		
	***Temperature Max***			23.6	9.9	33.6		
	***Temperature Min***			11.6	−4.1	27.5		

The bold values indicate a total of each variable

### Traffic outcomes

Table 2 presents a comparative analysis of the number of collisions, traffic light injuries, and KSI cases across Montréal, Québec, Laval, Longueuil, and Sherbrooke—from June through September 2015 to 2022. Montréal recorded a total of 44,698 collisions and 19,490 traffic light injuries, with 766 KSI cases. Under the 95th percentile heatwave definition, 1.16% of collisions and traffic light injuries, and 1.43% of KSI cases occurred. Using the 90th percentile definition, these percentages increased to 7.05%, 7.11%, and 5.48% for collisions, traffic light injuries, and KSI, respectively. Québec City had 20,675 collisions, 7,168 light injuries, and 560 KSI. Collisions and light injuries were slightly higher during the 95th percentile heatwave events (1.59% and 1.32%, respectively) compared to KSI cases (1.25%). The threshold-based 90th definition captured a higher percentage of incidents, with 6.42% of collisions, 6.35% of light injuries, and 5.89% of KSI. For Laval, which reported 10,173 collisions, 4,414 light injuries, and 165 KSI cases, the incidence of collisions and injuries during heatwaves by the 95th percentile definition was higher at 2.68% and 2.31%, respectively. Under the 90th percentile definition, the rate of collisions, injuries, and KSI rose to 6.31%, 6.28%, and 4.84%. In Longueuil, out of 35,705 total collisions, 3.42% correspond to the 95th percentile definition, whereas the 90th percentile definition accounted for 6.71% of collisions, 6.84% of injuries, and 6.26% of KSI cases. Lastly, Sherbrooke experienced 13,943 total collisions, 6,016 injuries, and 440 KSI cases. Here, the 95th percentile definition captured 4.51% of collisions and 4.87% of injuries. The 90th percentile recorded 6.91% of collisions, 7.54% of injuries, and 7.77% of KSI cases.

**Table 2:** Distribution of collisions, traffic light injuries, and KSI and heatwaves across selected Québec cities, June through September 2015–2022

City	Type of heatwave	Collisions	Traffic light injuries	KSI
***Montréal***		**N = 44,698**	**N = 19,490**	**N = 766**
	***Heatwave 90%***	3155 (7.05%)	1,386 (7.11%)	42 (5.48%)
	***Heatwave 95%***	522 (1.16%)	226 (1.16%)	11 (1.43)
**Québec**		**N = 20,675**	**N = 7,168**	**N = 560**
	***Heatwave 90%***	1,329 (6.42%)	455 (6.35%)	33 (5.89%)
	***Heatwave 95%***	328 (1.59%)	95 (1.32%)	7 (1.25%)
**Laval**		**N = 10,173**	**N = 4,414**	**N = 165**
	***Heatwave 90%***	641(6.31%)	277 (6.28%)	8 (4.84%)
	***Heatwave 95%***	273 (2.68%)	102 (2.31%)	5 (3.03%)
**Longueuil**		**N = 35,705**	**N = 16,467**	**N = 966**
	***Heatwave 90%***	2,396 (6.71%)	1,127 (6.84%)	58 (6.26%)
	***Heatwave 95%***	1,224 (3.42%)	532 (3.23%)	31 (3.38%)
**Sherbrooke**		**N = 13,943**	**N = 6,016**	**N = 440**
	***Heatwave 90%***	1133 (8.13%)	558 (9.28%	24 (5.45%)
	***Heatwave 95%***	629 (4.51%)	293 (4.87%)	11 (2.50%)

The bold values indicate a total of each variable

### Regression analysis

**Fig. 1:**
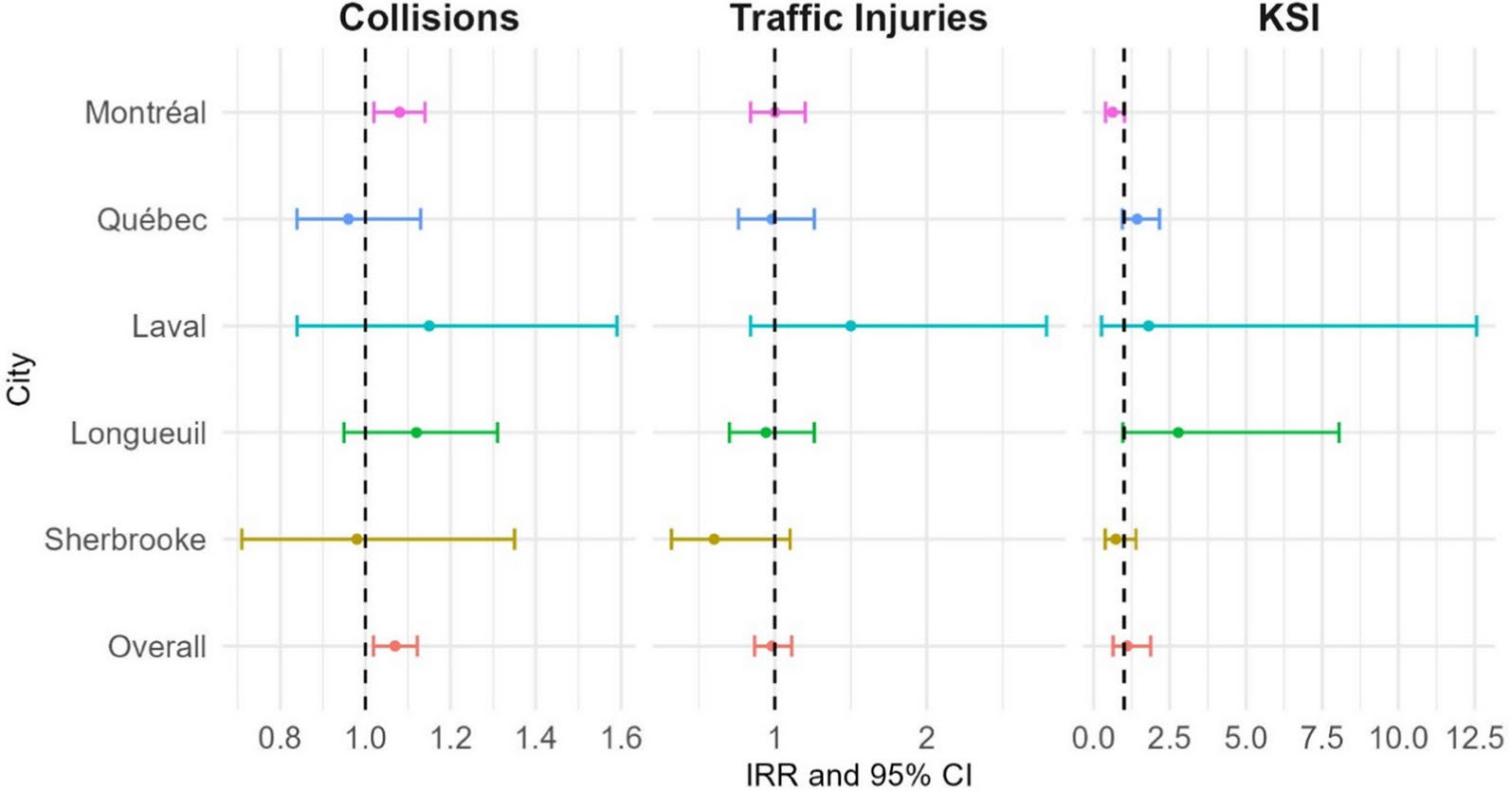
Associations of UHI, collisions, traffic light injuries and KSI rates per 100 000 population in selected Québec cities (June 1st- September 30th, 2015–2022): Results from Negative Binomial models. All models are adjusted by Heatwave 95%, COVID-19 Quebec Index, time and rainfall. KSI: Killed and Severely Injured. IRR: Incidence Rate Ratio. CI: Confidence Interval

Figure 1 summarizes the results from Negative Binomial models used to assess UHI and the three outcomes. In Montréal, UHI shows a modest increase in collisions (IRR: 1.08; 95% CI: 1.02–1.14), but the association with traffic injuries is not significant (IRR: 1.00; 95% CI: 0.84–1.20). The relationship is weaker for KSI,, with an IRR of 0.62 (95% CI: 0.39–1.01). In Québec, UHI is negatively associated with collisions (IRR: 0.96; 95% CI: 0.84–1.13) and traffic injuries (IRR: 0.98; 95% CI: 0.76–1.26), with a no significant positive association for KSI (IRR: 1.43; 95% CI: 0.94–2.17). Laval shows a no significant positive association with traffic injuries (IRR: 1.50; 95% CI: 0.84–2.79) and KSI (IRR: 1.81; 95% CI: 0.26–12.56). For collisions, the association is weaker (IRR: 1.15; 95% CI: 0.84–1.59). In Longueuil, UHI is positively but not significantly associated with collisions (IRR: 1.12; 95% CI: 0.95–1.31) or KSI (IRR: 2.78; 95% CI: 0.96–8.05). The association with traffic injuries is also not significant (IRR: 0.94; 95% CI: 0.70–1.26). In Sherbrooke, UHI shows no significant association with collisions (IRR: 0.98; 95% CI: 0.71–1.35), traffic injuries (IRR: 0.60; 95% CI: 0.32–1.10) and KSI (IRR: 0.73; 95% CI: 0.38–1.39). Overall, the 1.07 IRR for collisions (95% CI: 1.02–1.22) suggests a modest positive relationship with UHI across the cities. For traffic injuries, UHI is not significantly associated (IRR: 0.98; 95% CI: 0.87–1.11). Last, UHI has a mild positive association with KSI (IRR: 1.09; 95% CI: 0.64–1.88), but the wide confidence intervals indicate a level of uncertainty in the overall effect.

**Fig. 2:**
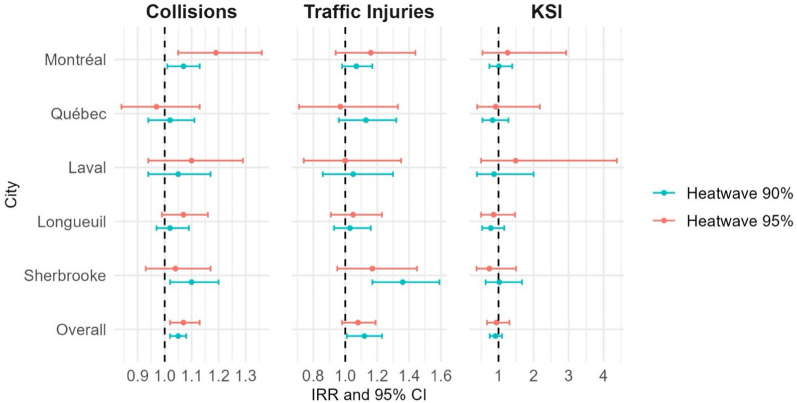
Associations of two definitions of heatwaves collisions, traffic light injuries and KSI rates per 100 000 population in selected Québec cities (June 1st- September 30th, 2015–2022): Results from Negative Binomial models. All models are adjusted by Urban Heat Island effect, COVID-19 Quebec Index, time and rainfall. KSI: Killed and Severely Injured. IRR: Incidence Rate Ratio. CI: Confidence Interval

Figure 2 summarizes the results from Negative Binomial models, respectively, used to assess the association between heatwave definitions (90th and 95th percentile) and the incidence of collisions, traffic injuries, and KSI cases, including an overall effect per heatwave. All models control for UHI, rainfall, seasonality, and the COVID-19 Quebec Index. In Montréal, both heatwave definitions show an increased incidence rate ratio (IRR) for collisions, with the 95th percentile definition showing a higher IRR (IRR: 1.19; 95% CI: 1.05–1.36). Traffic light injuries also show an increase with the 95th percentile definition (IRR: 1.16; 95% CI: 0.94–1.44), although the confidence interval suggests variability. For KSI, the IRR under the 95th percentile definition is higher (IRR: 1.26; 95% CI: 0.54–2.93) but with a wide confidence interval. Under the 90th percentile definition, the IRRs are lower, with collisions (IRR: 1.07; 95% CI: 1.01–1.13) and traffic injuries (IRR: 1.07; 95% CI: 0.98–1.17) showing modest increases. In Québec City, both heatwave definitions show little to no significant associations, with the IRRs close to 1.0 for collisions (IRR: 1.02; 95% CI: 0.94–1.11 for the 90th percentile and IRR: 0.97; 95% CI: 0.84–1.13 for the 95th percentile). Traffic light injuries (IRR: 1.13; 95% CI: 0.96–1.32 for the 90th percentile) show slight increases, but confidence intervals indicate considerable uncertainty. KSI shows no significant association across both heatwave definitions (IRR around 1.0). In Laval, collisions show a marginal no significant increase under the 95th percentile heatwave definition (IRR: 1.10; 95% CI: 0.94–1.29). Traffic light injuries and KSI show null associations, with KSI having an IRR of 1.49 (95% CI: 0.50–4.38). Under the 90th percentile definition, the IRRs for collisions and traffic injuries are 1.05 (95% CI: 0.94–1.17) and 1.05 (95% CI: 0.86–1.30), respectively, showing no strong associations. In Longueuil, the 95th percentile heatwave definition shows significant IRRs for collisions (IRR: 1.07; 95% CI: 0.99–1.16) and no significant for traffic light injuries (IRR: 1.05; 95% CI: 0.91–1.23), and KSI (IRR: 0.86; 95% CI: 0.50–1.47). The 90th percentile definition shows smaller no significant increases for collisions (IRR: 1.02; 95% CI: 0.97–1.09) and traffic light injuries (IRR: 1.03; 95% CI: 0.93–1.16). In Sherbrooke, the 90th percentile heatwave definition shows significant associations with both collisions (IRR: 1.10; 95% CI: 1.02–1.20) and traffic light injuries (IRR: 1.36; 95% CI: 1.17–1.59). The 95th percentile definition shows weaker no significant associations for collisions (IRR: 1.04; 95% CI: 0.93–1.17) and KSI (IRR: 0.74; 95% CI: 0.37–1.50). Overall, the 90th percentile heatwave definition shows a slight increase in the incidence rate ratio (IRR) for collisions (IRR: 1.05; 95% CI: 1.02–1.08) and traffic injuries (IRR: 1.12; 95% CI: 1.01–1.23). The IRR for KSI is not significant (IRR: 0.91; 95% CI: 0.75–1.10). The 95th percentile heatwave definition shows a more pronounced effect on collisions (IRR: 1.07; 95% CI: 1.02–1.13) and traffic injuries (IRR: 1.08; 95% CI: 0.98–1.19), with the association for KSI being weaker (IRR: 0.94; 95% CI: 0.67–1.31).

In Supplementary Material, Table S5 presents the I^2^ values and p-values for heterogeneity across cities. This information helps assess whether the associations between UHI and heatwaves and the three outcomes vary across the five cities. The results show that the UHI effect is highly heterogeneous (I^2^ > 60%) for KSI, and the heatwave 90% definition shows variability for traffic injuries (I^2^ > 60%). Tables S6, S7, and S8 present the Z-scores along with their associated p-values. In most cases, the IRRs across cities did not show significant differences at a p-value threshold of < 0.05. However, for collisions, Montréal exhibited a significantly higher IRR for the heatwave 95% definition compared to Québec (p = 0.04). For traffic injuries, Montréal showed a significantly lower IRR for the heatwave 90% definition compared to Sherbrooke (p = 0.01). Lastly, Montréal showed significantly lower IRRs than Québec (p = 0.01) and Longueuil (p = 0.01) for UHI when assessing KSI.

## Discussion

In this study, we analyzed the influence of UHI and heatwaves on road safety in five Québec cities (Montréal, Québec City, Laval, Longueuil, and Sherbrooke). Our objective was to assess whether these operationalizations yielded different impacts on road safety outcomes, including collisions, traffic light injuries, and KSI rates, using Negative Binomial models. The influence of high-temperature events on collisions, traffic injuries, and KSI demonstrates how environmental conditions may exacerbate vulnerabilities for road users. Previous studies have emphasized that extreme weather conditions, particularly high temperatures, negatively impact road users due to fatigue, reduced reaction times, and other physiological stressors [25, 41, 45]. Our study extends these findings by showing that these impacts may also be significantly influenced by UHIs.

Our analysis used two heatwave definitions based on the 90th and 95th percentile temperature thresholds, revealing varying associations with traffic outcomes. The 90th percentile heatwave definition generally captured a higher number of traffic incidents across all cities, as it included a broader range of days. In contrast, the more stringent 95th percentile threshold was linked to a modest yet statistically significant increase in collisions and traffic light injuries in Montréal and Longueuil. Montréal, in particular, has well-documented UHI effects, with temperatures consistently higher than in surrounding areas due to heat retention from buildings and limited vegetation [35, 46]. This constant exposure to elevated temperatures may explain why heatwaves, especially when defined at the 95th percentile, have a more pronounced effect on road safety in these cities. These findings align with studies from regions like Catalonia and Italy, where heatwaves were associated with 2.5% and 12% increases in crashes [3], respectively. However, comparisons across cities did not show significant differences using either heatwave definition, with the notable exception being Montréal compared to Québec City.

In cities with somewhat lower UHI conditions, such as Québec City and Sherbrooke, there were no significant associations between heatwaves and traffic outcomes at the 95th percentile threshold. Sherbrooke did show a notable association with traffic injuries at the 90th percentile. Given that no significant differences were observed across cities, these findings should be interpreted with caution, and the role of local environmental conditions, such as UHI effects, may warrant further investigation in future studies.

Acclimation—the adaptation of road users to increasing temperatures over time—also appears to be an essential factor when explaining outcomes’ variability across cities and heatwave thresholds. Our results align with Wu et al. [44], who suggested that acclimation to high temperatures might reduce the sensitivity of road users to extreme weather events, possibly dampening the effects of heatwaves on traffic outcomes. Our study's two heatwave definitions account for magnitude, helping to contextualize the role of acclimation in road safety. The relatively stronger associations between traffic outcomes and the 90th percentile definition across cities suggest that acclimation may mitigate some of the effects of extreme temperatures, as road users may become accustomed to warmer conditions as summer progresses. In contrast, the 95th percentile threshold represents sudden, intense temperature peaks that may surpass the acclimation threshold for many individuals, resulting in a higher incidence of traffic-related incidents.

Regarding traffic injuries and KSI outcomes, it is worth noting that the lack of significant findings in some cases, particularly for KSI and traffic injuries, may be attributed to smaller sample sizes. These smaller samples, especially in the context of specific cities or less frequent extreme temperature days, may reduce the statistical power to detect significant associations. Despite this, the trends observed for collisions, especially at higher temperature percentiles, suggest that heatwaves can still significantly influence road safety even with a more conservative definition.

Given the associations identified between heatwaves and road safety, ad-hoc urban road safety strategies should consider environmental factors like heatwaves and UHI effects. The overall effect of UHI across these five cities in increasing collisions by 7% suggests that these five cities may benefit from targeted interventions during heatwave periods. Measures such as expanding green spaces and implementing heat-reflective materials on roads and buildings [33] could reduce baseline temperatures, indirectly lowering the impact of heatwaves on road safety. Additional measures, such as public awareness campaigns about the dangers of driving, walking, or cycling in extreme heat conditions, including temporary reductions in speed limits or increased monitoring of high-risk areas, could mitigate some of the risks.

This study’s findings highlight the need to incorporate climate-resilient approaches within traffic safety planning frameworks, especially as climate change is expected to increase the frequency and intensity of heatwaves globally. For Québec and other regions experiencing high UHI effects, urban adaptation measures to address extreme temperatures and their impact on road safety should become a priority. These may include integrating adaptive infrastructure like heat-reflective pavements, increasing shade coverage along roadsides, or using cooling technologies in urban design [10], thereby reducing the need for temperature-related behavior adaptations among road users.

While our study advances critical insights into the relationship between heatwaves and road safety outcomes, there are some limitations to consider. Firstly, the reliance on a threshold-based definition of heatwaves inherently introduces variability in outcomes, as threshold sensitivity may differ across road users. Additionally, factors such as acclimation levels and varying socioeconomic profiles in different cities may also play a role in modulating the effects of heatwaves, potentially influencing how individuals respond to high temperatures. Although we included a proxy for the UHI effect to account for city-specific acclimation, the omission of more detailed demographic and socio-economic variables, such as income or education level, remains a limitation. These factors could further modulate how communities respond to heat exposure, making the inclusion of such variables an important direction for future research. The study was further limited by using aggregate data, which restricts the ability to account for individual-level variables like health status, age, or type of road users, all of which could impact individual’s vulnerability to extreme heat. A more detailed, individual-level analysis could offer valuable insights into how personal characteristics interact with environmental stressors to influence road safety outcomes. This is especially relevant when examining pedestrians and cyclists, who lack the protection of air conditioning usually found in motor vehicles, making them more vulnerable to extreme heat and other environmental conditions. Another limitation relates to the data itself, as the study spanned only the summer months (June–September) from 2015 to 2022. While this period allowed us to capture high-temperature events, expanding the dataset across other seasons could reveal additional patterns in the relationship between ambient temperatures and road safety outcomes. Future research should consider longer timeframes to account for year-round temperature variability and the impact of other extreme weather conditions, such as heavy rain and snowfall, on road safety. Additionally, the statistical power of our analysis was limited by the relatively small sample size, particularly for certain cities and variables like the KSI outcomes. While efforts were made to mitigate this by meta-analysis, which allowed us to pool estimates across cities, increasing the sample size or aggregating more cities could further improve statistical significance. Lastly, our study does not incorporate spatially granular analyses, such as those conducted at the street-link level or using semantic image segmentation methods, as proposed by Hu et al. [18, 19]. Such approaches could provide a richer understanding of how street-level built-environment features interact with climate variables to influence road safety outcomes. Future studies could build on these methodologies to explore how micro-level environmental factors contribute to traffic risks during extreme weather events.

Nonetheless, our findings open avenues for further research on the link between extreme weather events and road safety, particularly in the context of climate adaptation. This is particularly relevant as other studies should be carried out in cities where considerable effects of UHI have been reported. Future studies could also explore additional weather variables like humidity or air pollution, which may interact with temperature to influence road user behavior. Understanding the combined effects of multiple weather factors would provide a more comprehensive view of how climate variables affect traffic outcomes. Moreover, as climate change continues to shape weather patterns, research should also investigate the effectiveness of interventions designed to mitigate the impact of heatwaves on road safety. Such studies could examine the long-term effects of UHI reduction strategies on road safety outcomes, helping to inform urban planning decisions.

## Conclusion

Our study contributes to the growing body of literature on environmental and climatic factors and road safety by demonstrating how heatwaves and UHIs interact to impact traffic outcomes in five Québec cities. The significant associations between heatwave exposure and traffic incidents, particularly in cities with strong UHI effects like Montréal and Longueuil, underscore the importance of considering climatic factors in road safety planning. These findings suggest that targeted policy interventions and urban planning adjustments could play a crucial role in reducing heatwave-related road safety risks. As climate change intensifies, our study serves as a reminder that a climate-resilient approach to road safety is essential, not only for protecting drivers in extreme weather but also for building safer, more adaptable urban environments.

## Electronic supplementary material

Supplementary Material 1

## Declarations

### Availability of data and materials

The crash data that support the findings of this study are available *from Société de l'assurance automobile du Québec,* but restrictions apply to their availability, which were used under license for the current study and so are not publicly available. UHI [mean warm-season land surface temperature (LST)] was obtained from the Canadian Urban Environmental Health Research Consortium under the CANUE Data Use and Sharing Agreement. Meteorological data are available from the corresponding author upon reasonable request.
