# Supplementary material for: The influence of heatwaves on traffic safety in five cities across Québec with different thermal landscapes
Source: Inj Epidemiol. 2025 Feb 28;12:12. doi: 10.1186/s40621-025-00564-2 (PMC11869667; doi:10.1186/s40621-025-00564-2)

**Supplementary material**

**Table S1** Associations of UHI, collisions, traffic light injuries, and KSI rates per 100 000 population in selected Québec cities (June 1^st^- September 30^th^, 2015- 2022): Results from Poisson models.

|  | **Collisions** | | | **Traffic injuries** | | | **KSI** | | |
| --- | --- | --- | --- | --- | --- | --- | --- | --- | --- |
| **City** | **IRR** | **95%** | **CI** | **IRR** | **95%** | **CI** | **IRR** | **95%** | **CI** |
| **Montréal** | 1.08 | 1.01 | 1.14 | 1.00 | 0.89 | 1.13 | 0.63 | 0.39 | 1.01 |
| **Québec** | 0.94 | 0.82 | 1.08 | 0.97 | 0.77 | 1.21 | 1.37 | 0.90 | 2.08 |
| **Laval** | 1.12 | 0.94 | 1.48 | 1.65 | 0.95 | 2.86 | 1.46 | 0.26 | 8.25 |
| **Longueuil** | 1.10 | 0.93 | 1.30 | 0.92 | 0.70 | 1.21 | 2.87 | 0.97 | 8.50 |
| **Sherbrooke** | 1.00 | 0.75 | 1.32 | 0.59 | 0.32 | 1.09 | 0.68 | 0.45 | 1.01 |

All models are adjusted by the COVID-19 Quebec Index, heatwaves 95% and rainfall. KSI: Killed and Severely Injured. IRR: Incidence Rate Ratio. CI: Confidence Interval.

**Table S2** Associations of two definitions of heatwaves and collisions, traffic light injuries and KSI rates per 100 000 population in selected Québec cities (June 1^st^- September 30^th^, 2015- 2022): Results from Poisson models.

|  |  | **Collisions** | | | **Traffic injuries** | | | **KSI** | | |
| --- | --- | --- | --- | --- | --- | --- | --- | --- | --- | --- |
| **City** | **Heatwave** | **IRR** | **95%** | **CI** | **IRR** | **95%** | **CI** | **IRR** | **95%** | **CI** |
| **Montréal** | ***Heatwave 90%*** | 1.06 | 1.01 | 1.13 | 1.09 | 1.00 | 1.13 | 1.00 | 0.74 | 1.34 |
|  | ***Heatwave 95%*** | 1.19 | 1.05 | 1.36 | 1.23 | 1.00 | 1.53 | 1.12 | 0.55 | 2.31 |
| **Québec** | ***Heatwave 90%*** | 1.03 | 0.95 | 1.12 | 1.06 | 0.91 | 1.23 | 0.82 | 0.56 | 1.21 |
|  | ***Heatwave 95%*** | 0.96 | 0.81 | 1.12 | 0.88 | 0.64 | 1.24 | 0.82 | 0.33 | 1.98 |
| **Laval** | ***Heatwave 90%*** | 1.03 | 0.94 | 1.13 | 1.07 | 0.89 | 1.27 | 0.84 | 0.37 | 1.91 |
|  | ***Heatwave 95%*** | 1.10 | 0.94 | 1.30 | 1.03 | 0.75 | 1.42 | 1.42 | 0.49 | 4.09 |
| **Longueuil** | ***Heatwave 90%*** | 1.02 | 0.96 | 1.08 | 1.03 | 0.93 | 1.14 | 0.80 | 0.55 | 1.14 |
|  | ***Heatwave 95%*** | 1.07 | 0.99 | 1.16 | 1.05 | 0.91 | 1.22 | 0.89 | 0.56 | 1.43 |
| **Sherbrooke** | ***Heatwave 90%*** | 1.09 | 1.02 | 1.18 | 1.34 | 1.17 | 1.54 | 1.00 | 0.66 | 1.49 |
|  | ***Heatwave 95%*** | 1.05 | 0.95 | 1.17 | 1.15 | 0.95 | 1.40 | 0.74 | 0.35 | 1.54 |

All models are adjusted by the COVID-19 Quebec Index and rainfall. KSI: Killed and Severely Injured. IRR: Incidence Rate Ratio. CI: Confidence Interval.

**Table S3** Overdispersion Pearson and Deviance tests comparing Negative Binomial and Poisson models using heatwave 95% as only one independent variable

| **City** | **Outcome** | **Heatwave** | **Negative binomial models** | | **Poisson models** | |
| --- | --- | --- | --- | --- | --- | --- |
|  |  |  | *Over dispersion* | | *Over dispersion* | |
|  | *Collisions* | *95* | Pearson | Deviance | Pearson | Deviance |
| Montréal |  |  | 0.04 | 0.04 | 1.65 | 1.64 |
| Québec |  |  | 0.06 | 0.06 | 1.34 | 1.36 |
| Laval |  |  | 0.10 | 0.11 | 1.15 | 1.18 |
| Longueuil |  |  | 0.04 | 0.04 | 1.42 | 1.41 |
| Sherbrooke |  |  | 0.08 | 0.08 | 1.17 | 1.18 |
|  | Traffic injuries |  |  |  |  |  |
| Montréal |  |  | 0.09 | 0.10 | 1.89 | 1.90 |
| Québec |  |  | 0.22 | 0.26 | 1.78 | 1.84 |
| Laval |  |  | 0.32 | 0.43 | 1.72 | 1.84 |
| Longueuil |  |  | 0.13 | 0.11 | 2.03 | 1.99 |
| Sherbrooke |  |  | 0.25 | 0.30 | 1.71 | 1.77 |
|  | KSI |  |  |  |  |  |
| Montréal |  |  | 0.63 | 0.78 | 1.20 | 1.25 |
| Québec |  |  | 0.83 | 0.81 | 1.31 | 1.21 |
| Laval |  |  | 1.03 | 0.52 | 0.66 | 1.23 |
| Longueuil |  |  | 0.83 | 0.64 | 1.39 | 1.23 |
| Sherbrooke |  |  | 0.95 | 0.79 | 1.40 | 1.15 |

**Table S4** Overdispersion Pearson and Deviance tests comparing Negative Binomial and Poisson models using heatwave 90% as only one independent variable

| **City** | **Outcome** | **Heatwave** | **Negative binomial models** | | **Poisson models** | |
| --- | --- | --- | --- | --- | --- | --- |
|  |  | *90* | *Over dispersion* | | *Over dispersion* | |
|  | *Collisions* |  | Pearson | Deviance | Pearson | Deviance |
| Montréal |  |  | 0.04 | 0.04 | 1.64 | 1.66 |
| Québec |  |  | 0.06 | 0.06 | 1.34 | 1.36 |
| Laval |  |  | 0.11 | 0.12 | 1.15 | 1.18 |
| Longueuil |  |  | 0.04 | 0.04 | 1.42 | 1.41 |
| Sherbrooke |  |  | 0.08 | 0.08 | 1.16 | 1.17 |
|  | Traffic injuries |  |  |  |  |  |
| Montréal |  |  | 0.09 | 0.10 | 1.89 | 1.90 |
| Québec |  |  | 0.22 | 0.26 | 1.78 | 1.84 |
| Laval |  |  | 0.32 | 0.43 | 1.72 | 1.83 |
| Longueuil |  |  | 0.13 | 0.11 | 2.03 | 1.99 |
| Sherbrooke |  |  | 0.24 | 0.29 | 1.67 | 1.74 |
|  | KSI |  |  |  |  |  |
| Montréal |  |  | 0.63 | 0.78 | 1.10 | 1.23 |
| Québec |  |  | 0.83 | 0.81 | 1.31 | 1.21 |
| Laval |  |  | 1.03 | 0.52 | 0.66 | 1.23 |
| Longueuil |  |  | 0.83 | 0.64 | 1.39 | 1.23 |
| Sherbrooke |  |  | 0.95 | 0.79 | 1.40 | 1.15 |

**Table S5**. Test of Heterogeneity Results: City-Wise Variations in the Associations of Heatwaves and UHI with Collision, Traffic Injuries, and KSI

| **Exposures** | **Collisions**  I^2^, P-Value | **Traffic Injuries**  I^2^, P-Value | **KSI**  I^2^, P-Value |
| --- | --- | --- | --- |
| UHI | 0.00%, 0.57 | 0.02%, 0.34 | 66.62%, 0.02 |
| Heatwave 90% | 0.00%, 0.54 | 60.20%, 0.05 | 0.00%, 0.84 |
| Heatwave 95% | 0.02%, 0.33 | 0.00%, 0.77 | 0.00%, 0.79 |

**Table S6**. Z scores to assess differences in Incidence Rate Ratios of UHI definition across cities, using Montreal as a reference for collisions, traffic injuries, and KSI

| Outcomes | Montreal vs Quebec | | Montreal vs Laval | | Montreal vs Longueuil | | Montreal vs Sherbrooke | |
| --- | --- | --- | --- | --- | --- | --- | --- | --- |
|  | Z | *p* | Z | *p* | Z | *p* | Z | *p* |
| Collision | 1.45 | 0.14 | -0.38 | 0.70 | -0.42 | 0.68 | 0.58 | 0.56 |
| Traffic injury | 0.13 | 0.90 | -1.26 | 0.20 | 0.35 | 0.73 | 1.55 | 0.11 |
| KSI | **-2.58** | **0.01** | -1.05 | 0.9 | **-2.52** | **0.01** | -0.39 | 0.70 |

**Table S7**. Z scores to assess differences of Incidence Rate Ratios of heatwave 90% definition across cities, using Montreal as a reference for collisions, traffic injuries, and KSI

| Outcomes | Montreal vs Quebec | | Montreal vs Laval | | Montreal vs Longueuil | | Montreal vs Sherbrooke | |
| --- | --- | --- | --- | --- | --- | --- | --- | --- |
|  | Z | *p* | Z | *p* | Z | *p* | Z | *p* |
| Collision | 0.94 | 0.35 | 0.30 | 0.76 | 1.16 | 0.25 | -0.54 | 0.58 |
| Traffic injury | -0.58 | 0.56 | 0.16 | 0.87 | 0.53 | 0.60 | **-2.65** | **0.01** |
| KSI | 0.72 | 0.42 | 0.33 | 0.74 | 1.00 | 0.31 | -0.03 | 0.97 |

**Table S8**. Z scores to assess differences of Incidence Rate Ratios of heatwave 95% definition across cities, using Montreal as reference for collisions, traffic injuries and KSI

| Outcomes | Montreal vs Quebec | | Montreal vs Laval | | Montreal vs Longueuil | | Montreal vs Sherbrooke | |
| --- | --- | --- | --- | --- | --- | --- | --- | --- |
|  | Z | *p* | Z | *p* | Z | *p* | Z | *p* |
| Collision | **2.04** | **0.04** | 0.75 | 0.45 | 1.37 | 0.17 | 1.52 | 0.13 |
| Traffic injury | 0.92 | 0.34 | 0.79 | 0.43 | 0.74 | 0.46 | 0.74 | 0.46 |
| KSI | 0.51 | 0.61 | -0.24 | 0.81 | 0.74 | 0.46 | 0.95 | 0.34 |

**List of stations to obtain meteorological data per city**

**Montreal**

1. St Laurent (College)
2. Montreal Jean Brebeuf
3. Montreal Mcgill
4. Mctavish
5. Montreal Loyola
6. Cote St Luc
7. Poste Saraguay
8. Montreal Adac A
9. Montreal Intl A
10. Montreal/Pierre Elliott Trudeau Intl
11. Montreal Jar Bot
12. Montreal/Pierre Elliott Trudeau Intl A
13. La Salle
14. Cote-Ste-Catherine
15. Montreal Ice Control
16. Pointe Claire
17. Montreal-Est
18. Ste Genevieve
19. Auteuil
20. St Francois De Laval
21. St Constant
22. Montreal/St-Hubert
23. Montreal/St-Hubert A

**Quebec**

1. Domaine-De-Maizerets
2. Quebec
3. Beauport
4. Quebec 2
5. Lauzon (Aut)
6. St Romuald
7. Duberger
8. Ste-Foy (U. Laval)
9. Charlesbourg Parc Orlean
10. Courville
11. Villeneuve
12. Beauport
13. Les Saules
14. Courville De Poissy
15. Ste Foy
16. Cap Rouge
17. Ste Foy (Pie Xii)
18. Lauzon
19. Ste Foy Matapedia
20. La Courvilloise
21. St Jean Chrysostome
22. Charlesbourg Jar Zoo
23. Neufchatel
24. Quebec/Jean Lesage Intl
25. Loretteville
26. Quebec/Jean Lesage Intl A
27. Quebec Intl A
28. St Laurent Ile D'orleans
29. Beausejour

**Laval**

1. Laval Des Rapides
2. Ste Dorothee
3. Montreal Persillier
4. St Laurent (College)
5. Duvernay
6. Fabreville

**Longueuil**

1. St Lambert
2. Montreal Jar Bot
3. Ile Charron

**Sherbrooke**

1. Sherbrooke
2. Sherbrokke (University)
3. Lennoxville
4. Bromptonville

**Maps of the selected cities**

This information was obtained from Elections Canada

**Montréal**


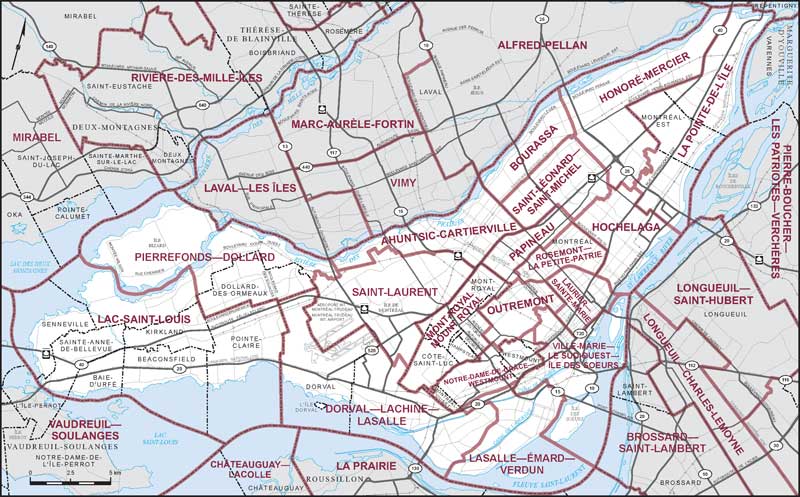


**Quebec**


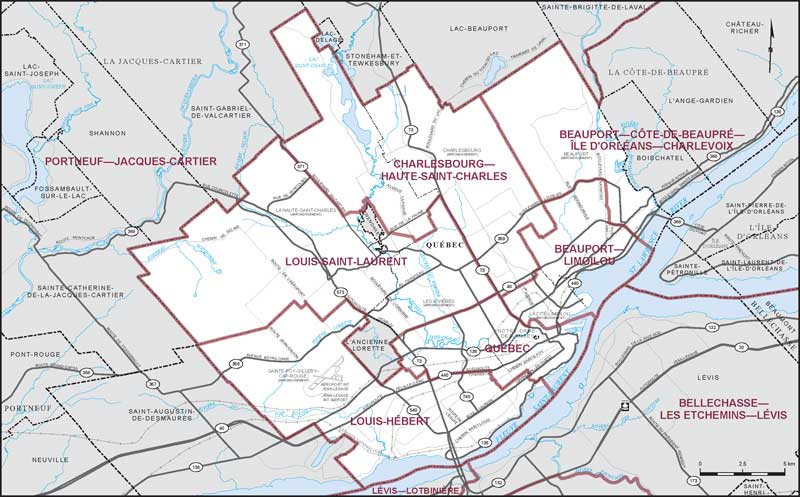


**Laval**


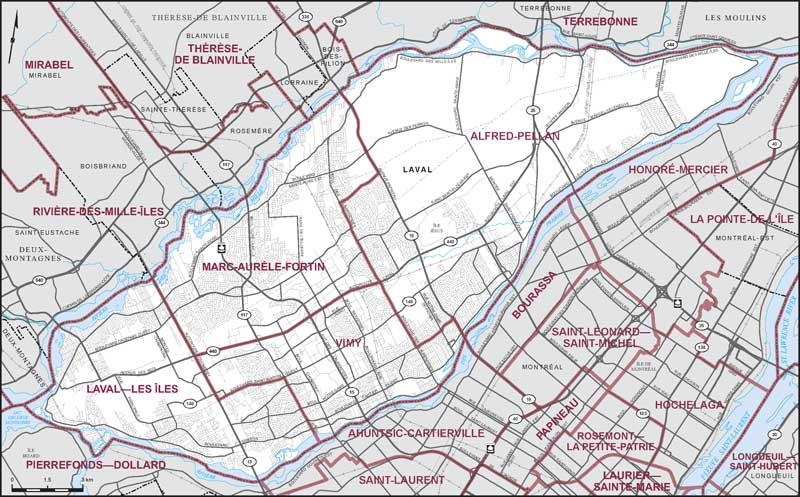


**Longueuil (Saint-Hubert)**


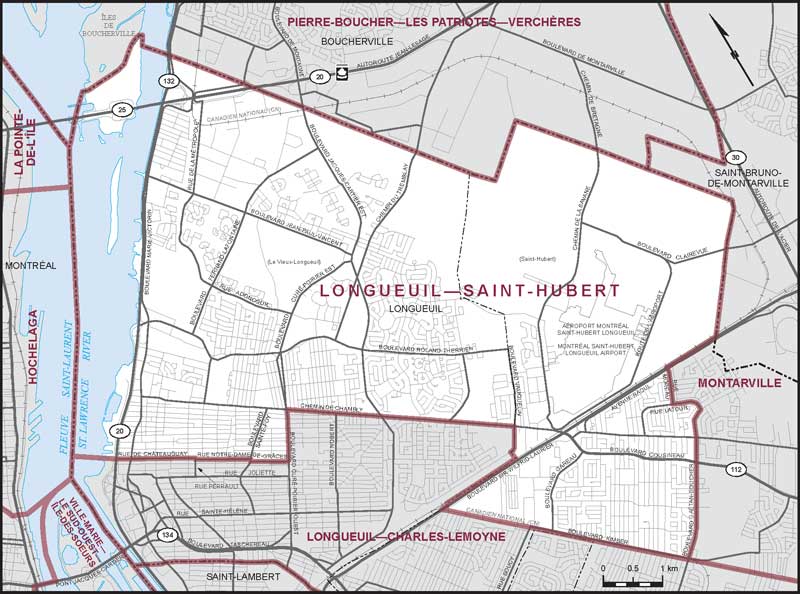


**Longueuil (Charles-LeMoyne)**


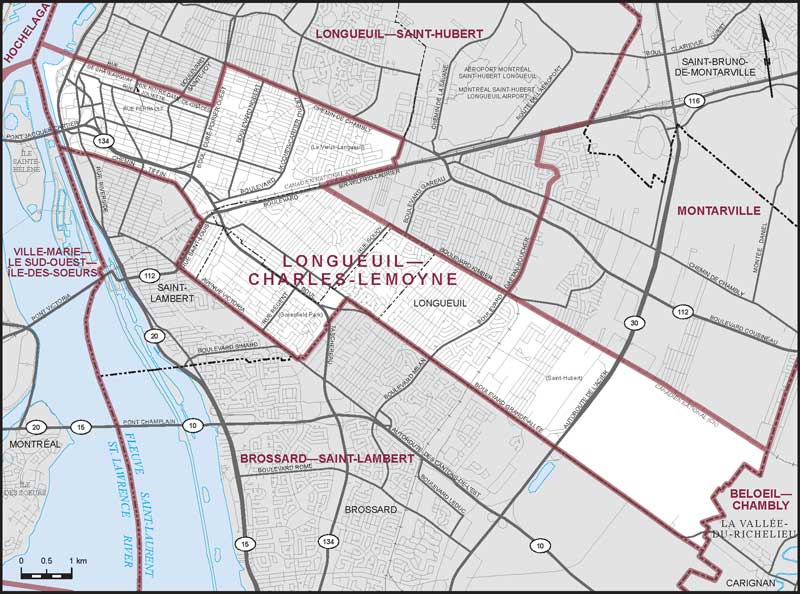


**Sherbrooke**


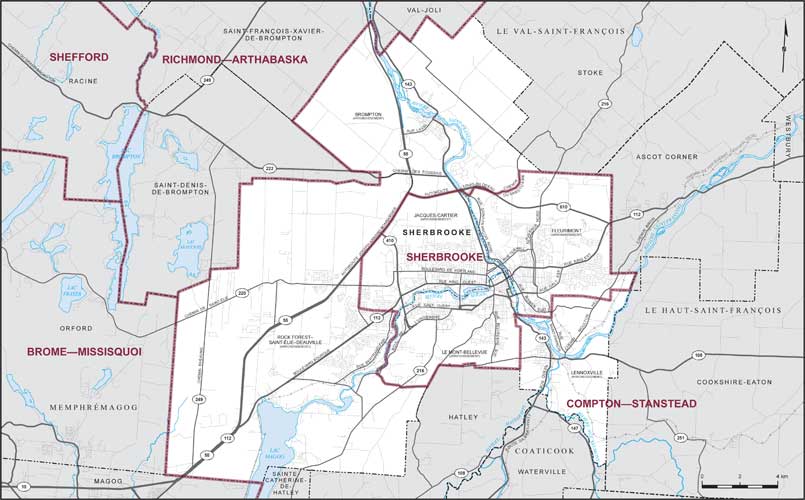

Supplement: Supplementary file 1 — Supplementary Material 1 [file 40621_2025_564_MOESM1_ESM.docx]
